# Complete Genome Sequence of the Novel Virulent Phage PMBT28 with Lytic Activity against Thermotolerant Salmonella enterica subsp. enterica Serovar Senftenberg ATCC 43845

**DOI:** 10.1128/genomeA.00568-18

**Published:** 2018-06-28

**Authors:** Sabrina Koberg, Erik Brinks, Viktoria Albrecht, Horst Neve, Charles M. A. P. Franz

**Affiliations:** a Department of Microbiology and Biotechnology, Max Rubner-Institut, Federal Research Institute of Nutrition and Food, Kiel, Germany

## Abstract

The complete genome sequence of the novel virulent Siphoviridae phage PMBT28, which may potentially be utilized for the biocontrol of Salmonella spp., is reported here. The phage was isolated using Salmonella enterica subsp. enterica serovar Senftenberg ATCC 43845 as a bacterial host. The genome had no significant sequence similarity to known phage genomes.

## Abstract (precis)

The complete genome sequence of the novel virulent Siphoviridae phage PMBT28, which may potentially be utilized for the biocontrol of Salmonella spp., is reported here. The phage was isolated using Salmonella enterica subsp.

## GENOME ANNOUNCEMENT

Salmonella enterica subsp. enterica serovar Senftenberg ATCC 43845 is a thermotolerant pathogen (1–3), which was originally isolated from dried eggs in China (4). It has been associated with foodborne disease outbreaks, as well as infections of burn wounds, and was associated with acquired multidrug resistance phenotypes (5). Bacteriophages, the natural enemies of bacteria, represent a possible alternative for antibiotics, as they target bacteria specifically at the species or strain level.

For our study, we isolated the virulent bacteriophage PMBT28 from a municipal sewage treatment plant in Germany using S. Senftenberg. By transmission electron microscopic analysis, the phage (negatively stained with 2% [wt/vol] uranyl acetate) was assigned to the Siphoviridae family, with a long, but thin, noncontractile tail (length, 115.1 ± 4.0 nm, width, 10.2 ± 0.6 nm) and an isometric head (diameter, 59.9 ± 1.5 nm) (*n* = 5). DNA was isolated from 2 ml high-titer lysate using a phage DNA isolation kit (Norgen Biotek, Thorold, Canada) and quantified using a Qubit 3.0 fluorometer (Invitrogen, Karlsruhe, Germany). For genome sequencing, the Nextera XT DNA library preparation kit and the MiSeq reagent nano kit v2 were used according to the manufacturer’s instructions using a MiSeq sequencer (Illumina, Munich, Germany). A total of 48,530 paired-end reads (2 × 251 bp) were generated, from which 47,953 reads were *de novo* assembled into a single contig with a total length of 48,113 bp and a G+C content of 58.6 mol% using Geneious version 9.1.2 (6) and SPAdes (7). The assembled genome had a high (228-fold) coverage. Annotation was performed automatically with the Rapid Annotations using Subsystem Technology (RAST) server (8), followed by manual comparison of predicted open reading frames (ORFs) with proteins using the databases BLASTP (9) and SMART (10). Among the predicted 68 ORFs, ORFs coding for phage-specific proteins could be identified, e.g., for a phage small and large terminase subunit (ORF65 and ORF1), a capsid protein (ORF5), tail fiber proteins (ORF6, ORF19, and ORF21), a tape measure protein (ORF15), and a minor tail protein (ORF20). In addition, ORF66 specifies a phage holin belonging to superfamily III, with three transmembrane domains (11). Although conserved phage proteins were found, no functions could be determined for the majority (i.e., 74%) of the ORFs. Structural analysis of the terminase large subunit with SMART (10) indicated a conserved domain, which has also been identified in *pac*-type phage E. coli T4 (12). Moreover, the circularly assembled genome indicated an absence of defined ends, suggesting that PMBT28 is a *pac*-type phage. Therefore, position 1 of the genome was arbitrarily selected at the first nucleotide of the predicted terminase large subunit gene (ORF1). Using tRNAscan-SE version 2.0 (13), no tRNAs could be identified. Analysis with PHACTS (14) revealed no lysogeny-related genes, thus confirming the lytic lifestyle of PMBT28. No genes encoding acquired antibiotic resistance were detected using ResFinder (15). A megablast (9) search indicated that the genome showed only similarity to a notably short fragment of the Dickeya paradisiaca Ech703 genome (GenBank accession no. CP001654). In conclusion, phage PMBT28 represents a novel phage with the potential to be used as a biocontrol agent for food safety applications.

### Accession number(s).

This complete genome sequence has been deposited at GenBank under the accession no. MG641885.
